# Interhemispheric axonal sprouting occurs after pial removal in mice

**DOI:** 10.1038/s41598-024-75278-4

**Published:** 2024-10-21

**Authors:** Kasra Nikouei, Leonardo Garma, Fatima Memic, Jens Hjerling-Leffler, Ezequiel Goldschmidt

**Affiliations:** 1 Division of Molecular Neurobiology, Department of Medical Biochemistry and Biophysics, Karolinska Institutet, Stockholm, Sweden; 2 Breast Cancer Clinical Research Unit, Centro Nacional de Investigaciones Oncologicas–CNIO, Madrid, Spain; 3 Department of Neurosurgery, UCSF Weill Institute for Neurosciences, 400 Parnassus Ave, Suite A808, San Francisco, CA 94143 USA

**Keywords:** Neuroplasticity, White matter injury, Cortico–cortico transpial bypass, Mouse model, Biological techniques, Neuroscience, Neurological disorders

## Abstract

White matter lacks the kind of plasticity that is present in the cortex, and subcortical injuries often result in permanent neurological deficits. Because cortical regions share common subcortical nuclei, creating new intergyral connections may allow for the bypass of subcortical damage. In this manuscript, a surgical interhemispheric bridge is created in mice, providing a model for an intercortical transpial bypass. To model this bypass, a midline craniotomy followed by interhemispheric (IH) pial removal was performed in C57BL/6 mice, allowing for the juxtaposition of the right and left prefrontal cortices. Adeno-associated virus (AAV) expressing tdTomato under a neuronal-specific promoter were injected into the right hemisphere. Animals were sacrificed two and four weeks after surgery, and axonal sprouting and glial changes were assessed in the “bypass” (BP) operation and sham surgery. Surgery did not result in any clear functional impairments. Removing the pia resulted in the formation of a physical connection between the hemispheres and the loss of the normal pial IH barrier. Cortical layer I became thinner with neuronal bodies in closer proximity than in the sham group. New interhemispheric axonal crossings were visible at two and four weeks in the BP group but not in the sham mice. These findings constitute the first step in the development of a cortico–cortico transpial bypass, allowing us to test a new way to surgically restore neurological function.

**Supplementary Information:**

The online version contains supplementary material available at 10.1038/s41598-024-75278-4.

## Introduction

Common neurological disorders including stroke, trauma, and demyelinating diseases cause subcortical damage^1–7^, impairing normal communication to and from healthy cortical regions and resulting in loss of function. So far, efforts to restore tissue after white matter tract injury have been focused on repairing the damaged area with stem cells, growth factors, and scaffolds^8–10^. However, the neuroplastic processes described for the cortex are not present to the same degree in the white matter, and recovery from white matter injury often results in more permanent damage than limited cortical lesions^11–13^.

**Fig. 1:**
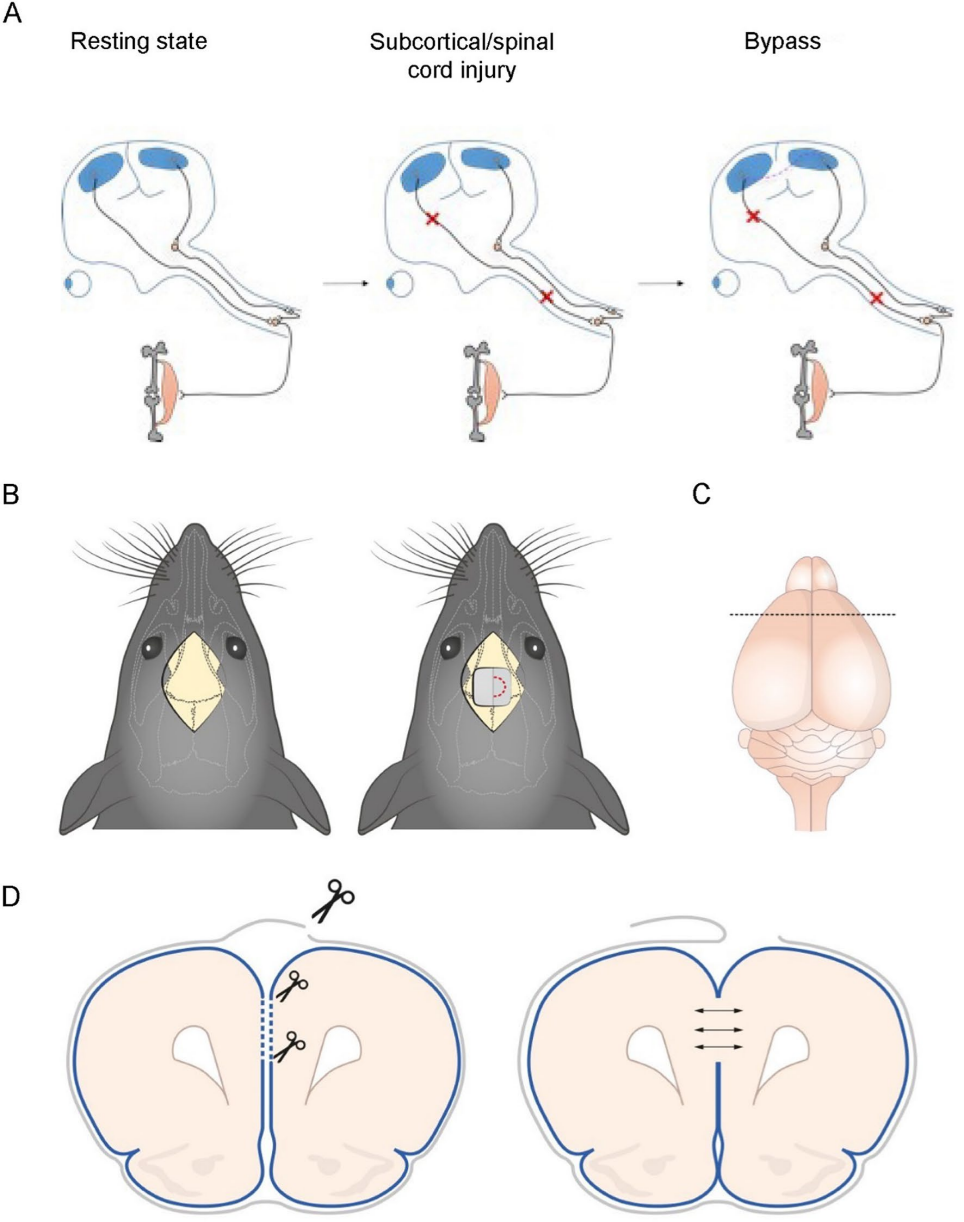
Theoretical benefits of a cortical bypass and experimental design. (**A**) Corticospinal tract axons end in the anterior horn and are also connected to other long tracts such as the vestibulospinal and the rubrospinal tracts (resting state). When the corticospinal tract is interrupted, the central gyrus neurons cannot reach the spinal cord (subcortical injury). In this example, neurons from the central gyrus would theoretically reroute their output through other unaffected tracts (bypass). (**B**) Depicts the surgical technique; a midline craniotomy of 2 × 3 mm over bregma is performed, the dura is opened in a “C” fashion and the sagittal sinus is dissected and displaced laterally. A sharp arachnoid knife is used to open coincidental pial windows on each hemisphere, allowing the neural tissue to expand medially, contacting the contralateral hemisphere and creating a bridge. (**C**) Illustration of the craniotomy site showing the position of the sagittal sinus, which was dissected and retracted, exposing the interhemispheric fissure, and allowing us to remove the pia in adjacent cortical regions shown in (**D**).

Brain folding places functionally distinct neurons from adjacent gyri in close proximity; separated by only two pial layers, and almost in contact in the Euclidean space^14–16^. Adjacent gyri have distinct subcortical connections but share relay stations, thus, directly connecting adjacent gyri to reroute information could present novel therapeutic approaches for white matter tract injuries (Fig. 1A)^17,18^. This approach could harness the plasticity of the cortex to create an alternative pathway to bypass subcortical injury.

Substantial integration through direct connectivity across sensory modalities is proof that functionally different neurons communicate with each other, although recent studies suggest that connectivity via subcortical structures is more important for circuit integration^19–22^. The rules governing the formation and plasticity of these direct connections are not well understood, and it is possible that the formation of new cortical connections could increase plasticity.

In this manuscript, we ask whether we can create a neural bridge between two cortical areas by removing the pial layers between brain hemispheres in a murine model. This model allowed us to test whether this novel cortical bypass procedure results in neurite crossings between brain hemispheres.

## Methods

### Animals

30–45 days old C57BL/6 wild-type mice (Janvier labs) were used. Two to five mice were housed per cage with a 12-h light–dark cycle. Both male and female mice were used for experiments. All mice had ad libitum access to water and food. Mice were randomly assigned to the “Bypass” (BP), or sham surgery and all cages contained mice with both treatments. Either treatment was done via the same incision and appeared the same after surgery. Animals were sacrificed two or four weeks after surgery. All animal handlings were in accordance with the relevant guidelines, regulations and protocols approved by the local ethical committee for experiments on laboratory animals (Stockholms Norra Djurförsöksetiska nämnd, Sweden), and reported in compliance with the ARRIVE guidelines.

### Anesthesia and surgery

Mice were kept anesthetized with 1.5% isoflurane (IsoFlo, Zoeteis) during the virus injection, the BP/sham operation and before cervical dislocation. For surgery, the mice were placed on a 37°C electric heating pad (Harvard Apparatus), and we applied ointment on the eyes (Dechra). After placing the mice on the stereotactic frame (Kopf Instruments) we sterilized the skin of the skull with Klorhexidin (Fresenius Kabi) and then ectopically applied 1% lidocaine (Hi-tech Pharmacal). A 1.5 cm midline incision exposing the coronal and sagittal suture was made and the skin flaps retracted with stiches (Fig. 1B). Injection with ssAAV-5/2-hSyn-chl-tdTomato-WPRE-SV40p(A), expressing tdTomato under the neuron-specific synapsin promoter, was done via a 2 mm circular craniotomy with a drill (Foredom MH-170) 2 mm anterior to bregma (2 mm antero-posterior axis), 2 mm lateral of the midline (2 mm mediolateral axis) and 2 mm deep from the pia (dorso-ventral axis). A total of 0.5 μl of (2 × 10^12^ viral molecules per ml) was then injected with a glass micro-pipette and a stereotactic injector (Nanoliter 2000, World Precision Instrument). The virus was delivered at a rate of 0.025 μl per minute. The pipette was retracted five minutes after the bolus was delivered. Fifteen minutes after viral injection, a 2 × 3 mm craniotomy over bregma was performed using a hand-held high-speed drill. The dura was then opened on both sides of the superior sagittal sinus. For the sham mice, this was the end of the surgery, and the skin was closed. For the BP mice, the IH fissure was dissected protecting the sinus and under the microscope using a sharp micro dissector the pial layers of both hemispheres in the prefrontal cortex were gently removed allowing direct contact between the right and left cortices, forming a tissue bridge (Fig. 1C-D, Supplementary Fig. 1). Hemostasis was obtained and the incision was closed. Before the operation analgesia (5 mg kg − 1 Carprofen) was administered subcutaneously and repeated 24 and 48 h after surgery.

### Tissue collection

Two or four weeks after surgery and after deep anesthesia, transcardial perfusions were performed with ice-cold phosphate buffer (PBS) followed by 4% paraformaldehyde (PFA)/PBS solution. The brains were carefully dissected and post-fixated for three hours on ice in 4% PFA/PBS solution. The brains were rinsed and cryo-protected in 30% sucrose/PBS solution for 48 h. The brains were embedded in optimal cutting temperature (O.C.T) compound (Tissue-Tek) and frozen on dry ice. The frozen brains were kept in -80 °C until usage and cryo-sectioned in 16 μm sections with a cryostat (Cryostar); sections were kept in -80 °C until staining protocol.

### Immunohistochemistry

The tissues were washed with PBS for 10 min. They were then blocked for one hour with blocking buffer (1.5% normal goat serum and 0.1% Triton X-100 in PBS) at room temperature. They were incubated with the primary antibodies overnight at 4 °C. After washing with PBS three times for 10 min each at room temperature, the tissues were incubated in secondary antibodies conjugated with Alexa fluorescent dyes 488, 555 and 647 (1:1000, Molecular Probes) for one hour at room temperature. The tissues were washed three times for 10 min each with PBS at room temperature, followed by DAPI nuclear staining. They were then covered with mounting medium and glass coverslips.

### Data collection and analysis

Images were acquired with Zeiss LSM800 and LSM800-airy fluorescent microscope. All analyses were performed on a defined optical area centered on the interhemispheric fissure, measuring 500 µm in the direction perpendicular to the fissure and 600 µm in the other axis. The position of the fissure was annotated automatically for each point in the direction along its length (the x axis on the image) using the DAPI signal. First, the “y” coordinate of maximum DAPI intensity in a band of 1000 pixels (123.5 µm) around the vertical center of the image was found for each x coordinate. Then a rolling window of 1000 pixels was applied to these maxima, and the position of the fissure at each point was estimated as the median within the window. Cells were detected automatically using the DAPI signal and the Python implementation of the Open Source Computer Vision Library (OpenCV, version 4.5.5.64)^23^. First, the images were binarized using Otsu’s method^23^. Then erosion followed by dilation was applied with a 10 × 10 pixel kernel to remove isolated pixels obtained in the binarization process. The distance to the nearest zero-valued pixel was computed for each pixel with a value of 1 using the Scipy library (version 1.8.1)^24^. Local maxima were detected on the distance map, requiring a minimum distance of 20 pixels between them. Then, a putative cell label was assigned to each maximum and all its connected components. Finally, the labeled elements were segmented using the watershed algorithm implemented in the scikit-image library (version 0.19.2). Gfap + cells were detected using the same approach on the Gfap signal intensities rather than the DAPI.

The NeuN intensities of individual cells were estimated as the mean intensity within the bounding box of the automatically detected cells on the DAPI channel. Cells were considered NeuN positive if this value was above 20% of the maximum possible NeuN intensity. The distribution of cell positions was estimated using the Gaussian kernel density estimation implemented in the Scipy library (version 1.8.1). The location of the fissure was estimated using the Iba1 signal. First, the signal was smoothed using a Gaussian blur with a box size of 200 pixels (24.71 µm). Then the maximum intensity point was calculated for each y coordinate. The fissure location was estimated as the median of a rolling average of 500 pixels over the maxima.

All data is presented as mean ± standard deviation (SD), and statistical significance was with a two-tailed t-test if not stated otherwise. For the experiments assessing pial changes, five animals per treatment were used at two different time points (20 total). For experiments assessing neural crossings, a different set of five animals per treatment per time point was used (also 20 in total). Within those groups, a subgroup of samples was used for different analyses.

## Results

BP and sham operations did not produce any visible neurological deficits during wellness checks or weight divergence. Mice were assessed twice per day by the animal facility staff and researchers.

### Pial removal yields a tissue bridge with extensive remodeling of the pial barrier and cortical layer I

**Fig. 2:**
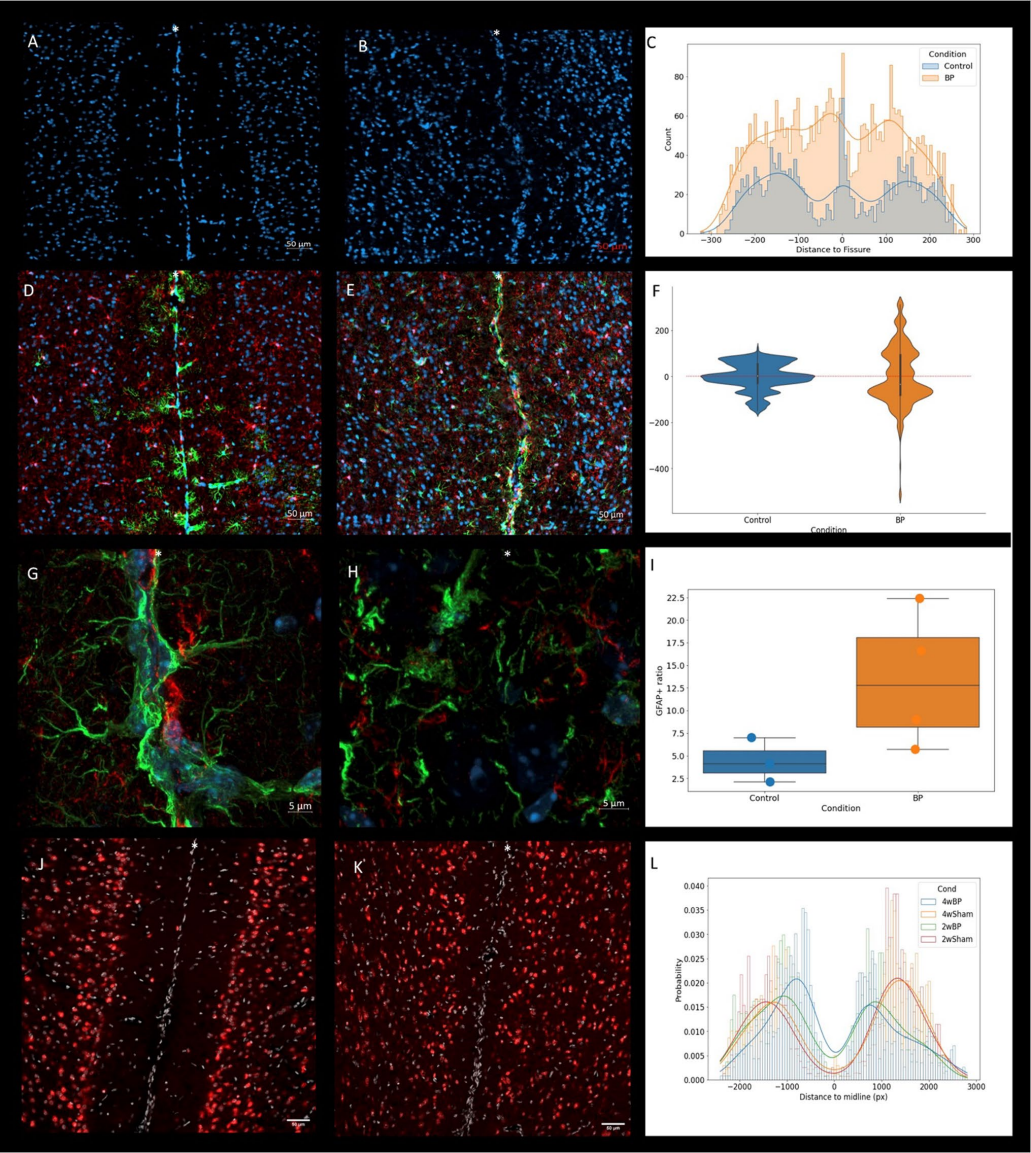
Pial removal modifies the normal cellular anatomy of the interhemispheric fissure. (**A**) Representative image of DAPI-signal in a 5 × magnification image of the interhemispheric fissure showing the normal pial epithelium at the midline and the normal architecture of cortical layer I in a sham animal. (**B**) Same as in A. but in BP animal four weeks after BP surgery. The normal pial epithelium is replaced by a hypercellular response resulting in curving of the interhemispheric interface, indicating loss of the normal pial barrier. (**C**) The cellular distribution from the midline in BP and sham conditions. Sham animals exhibit a “W” distribution with a peak at midline corresponding to the pia, low cell counts on layer I followed by higher cellularity deeper in the cortex. The BP surgery redistributed the cells in the area of interest with a valley close to the midline but deviated to the right, and peaks on either side corresponding to a higher cellular layer I. (**D**) (sham) and (**E**) (4-week BP) astrocytes are marked with Gfap (green) and microglia with Iba1 (red). The astrocytic pial processes mark an intact pial barrier that is remodeled after the pia is removed; astrocytes in (**E**) lose their normal architecture and exhibit hyperchromatic nuclei. (**F**) The distribution of the Iba1 signal relative to the midline is quantified, illustrating the differences in microglia distribution between sham and BP animals; the midline is marked with 0, and numbers express deviation from the midline to the right (+) and left (−). (**G**) (sham) and (**H**) (4-week BP) show representative images of the IH fissure at a higher magnification (Gfap, green; Iba1, red). Sham animals exhibited normal lining astrocytes at the cortical edge, which are lost after the BP surgery, in which the midline is not histologically visible. **I.** Changes in the glial composition at the area of interest, shown as the ratio of Gfap/total fluorescence ratio, showing a higher proportion of astrocyte marking for BP animals. (**J**) (sham) and (**K**) (4-week BP) show the distribution of neuronal nuclei (NeuN, red). (**L**) The BP surgery resulted in an increased number of neuronal nuclei closer to the interhemispheric fissure, where the proportion of NeuN + nuclei is plotted vs. the distance to the physical midline. The physical midline is marked with an asterisk.

The BP surgery effectively removed the pia and altered the configuration of cortical layer I (Supplementary Fig. 2). Pial removal resulted in direct contact between the ‘naked’ right and left cortices with increased cell density at the midline and reduced width of layer I**.** Sham animals had a high nuclear density at the midline, corresponding to the pia, while the BP protocol resulted in the loss of the organized pial layer with a multicellular response at the surgical site (Fig. 2A-C).

Cell number within the region of interest increased in the BP protocol compared to sham animals (sham 637.67 ± 115.57, BP 846.25 ± 34.83, *p* value < 0.05, n = 4 per group). The distribution of cells around the fissure in the sham cases showed a structure with three maxima: one centered on the IH fissure (mean distance to fissure 0.552 ± 3.71 µm) and two on either side, 150.12 ± 9.9 µm and 150.84 ± 13.82 µm away from the fissure. The estimated distribution of distances from the fissure on the BP cases was different from the sham mice, containing either two or three maxima (Fig. 2C). In the cases where three maxima were detected, the central one was displaced from the IH fissure by 25.6 ± 5.7 µm (*p* value < 0.05, n = 4 per group). In summary, the BP protocol increased the cellularity close to the IH fissure, replaced the pial bilayer with a multicellular response, and resulted in a solid newly formed bridge between hemispheres.

### The BP protocol remodeled the pial organization and structure around the IH fissure

The location of the IH fissure was estimated using the microglia marker Iba1 signal intensity (see methods; Fig. 2D-F). The deviation of the fissure was computed as the difference between the putative fissure location at each “y” coordinate and the mean fissure location on the whole image (Fig. 2F). The absolute deviation of the fissure was increased in the BP mice (sham 46.45 ± 17.14 µm, BP 102.64 ± 28.8 µm, *p* value < 0.05, n = 3 per group). This further showed that the BP surgery was effective in removing the normal pial barrier between the right and left hemispheres. In addition, the usual straight line formed by the IH fissure was replaced with a curved edge between hemispheres.

Astrocytes lining the pia, marked by Gfap expression, were present in sham mice, whereas the BP surgery resulted in loss of pial processes and modified the cellular composition of the IH fissure. After pial removal the IH fissure was composed of an alternative bridging tissue in which normal midline structures such as a pial epithelium and specialized glia were no longer present (Fig. 2D-E, G-H). The percentage of Gfap positive cells at the IH was seemingly increased in the BP cases, however just above our significance threshold (sham 4.4 ± 2.01%, n = 3; BP 13.43 ± 6.52%, n = 4; *p* value = 0.06; Fig. 2I).

### Pial removal approximated right and left cortical neuronal bodies

Neuronal bodies were marked with NeuN. Two weeks after BP surgery the neurons were significantly closer to midline (sham 2791.65 ± 247.68 µm; BP 2021.83 ± 153.15 µm; *p* value < 0.05, n = 3 per group) than their sham counterparts and even closer four weeks after BP surgery (sham 2707.41 ± 97.81 µm; BP 1636.133 ± 137.71 µm, n = 5 per group; *p* value < 0.05; Fig. 2J-L).

Altogether, the BP surgery resulted in a glial and neuronal re-organization providing a scaffold for neurites to grow between brain hemispheres.

### Interhemispheric sprouting of neuronal processes occurs after pial removal

**Fig. 3:**
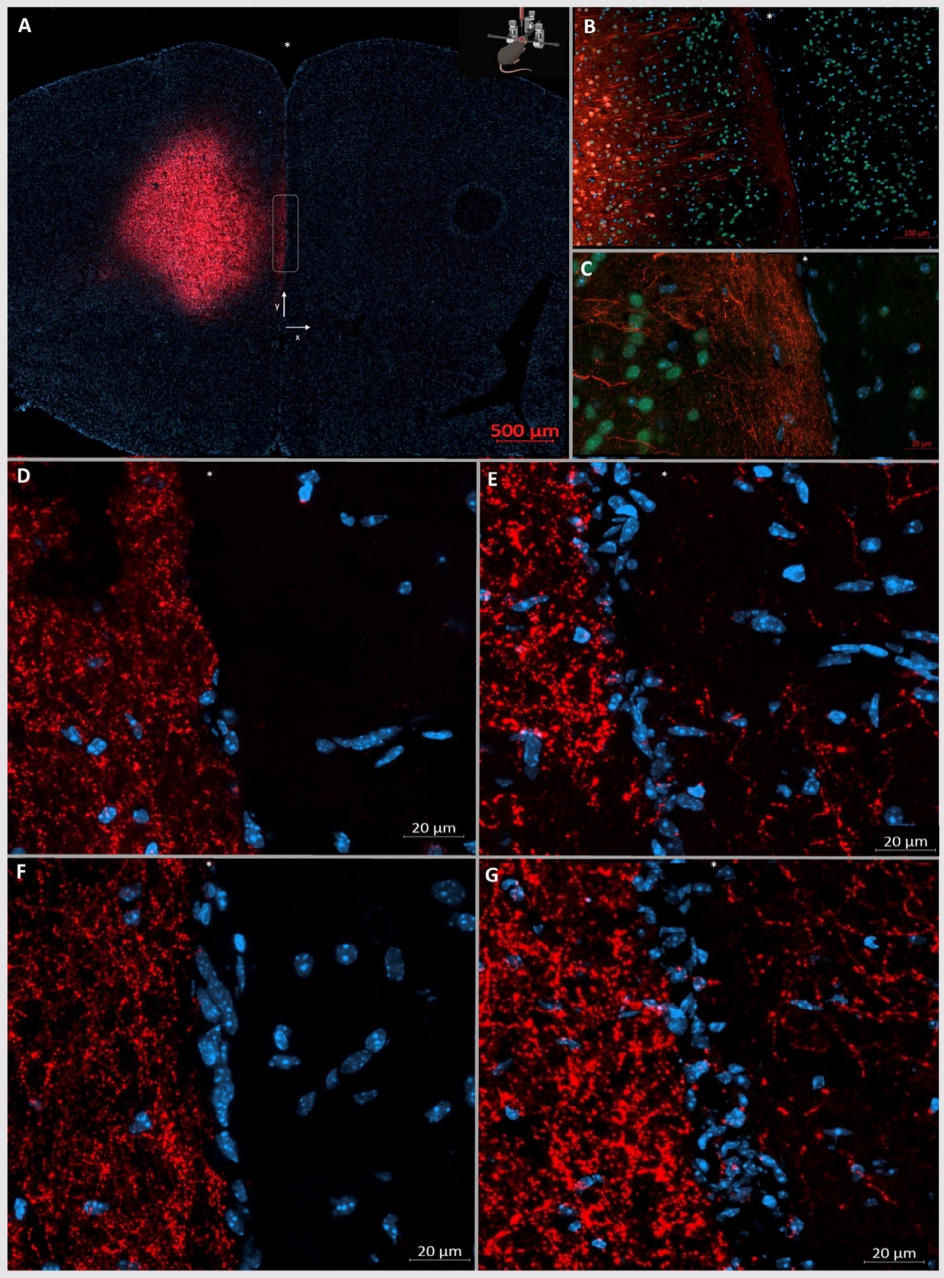
Pial removal in the interhemispheric fissure results in neurite crossing 2 and 4 weeks after surgery. (**A**) TdTomato expression after ssAAV-5/2-hSyn-chl-tdTomato-WPRE-SV40p(A) injection into the right hemisphere targeting the premotor cortex. (**B**) TdTomato signal (red) in a sham animal showing the injection site 2 mm deep from the pial surface (5 × objective), NeuN in green, and DAPI in blue. (**C**) as in B, but with 20 × objective. Scarce tdTomato signal can be seen on the left (non-injected) hemisphere corresponding to callosal fibers. (**D**) and (**F**) show representative images two and four weeks after sham surgery, respectively. The injected hemisphere shows tdTomato-positive neurites reaching the pia and tdTomato-negative contralateral hemisphere**.** (**E**) and (**G**) show representative images two and four weeks after BP surgery, respectively. After pial removal neurite crossings are visible through the tissue bridge across the interhemispheric fissure in BP mice. The white box in (**A**) shows the magnified area in other panels and figures. The x and y axes are defined for quantitative analysis. The physical midline is marked with an asterisk.

To assess whether the BP surgery resulted in crossings of neurites, we injected the premotor cortex of one hemisphere with an AAV expressing tdTomato under the neuron-specific promoter, synapsin. To reduce the risk of spillage over to the contralateral hemisphere, injections were performed 2 mm lateral from the midline (Fig. 3A). Neurites from deep cortical regions to ipsilateral cortical layer I can be seen two weeks after injection (Fig. 3A-C). Two and four weeks after surgery, none of the animals subjected to the sham protocol exhibited neurite crossings between the left and right hemispheres (Fig. 3D, F). In contrast, all animals in which the BP protocol was applied exhibited multiple tdTomato-expressing crossing neurites (Fig. 3E, G). Weak tdTomato signal could be seen on the non-injected hemisphere due to the crossing of transduced callosal fibers in both sham and BP mice.

**Fig. 4:**
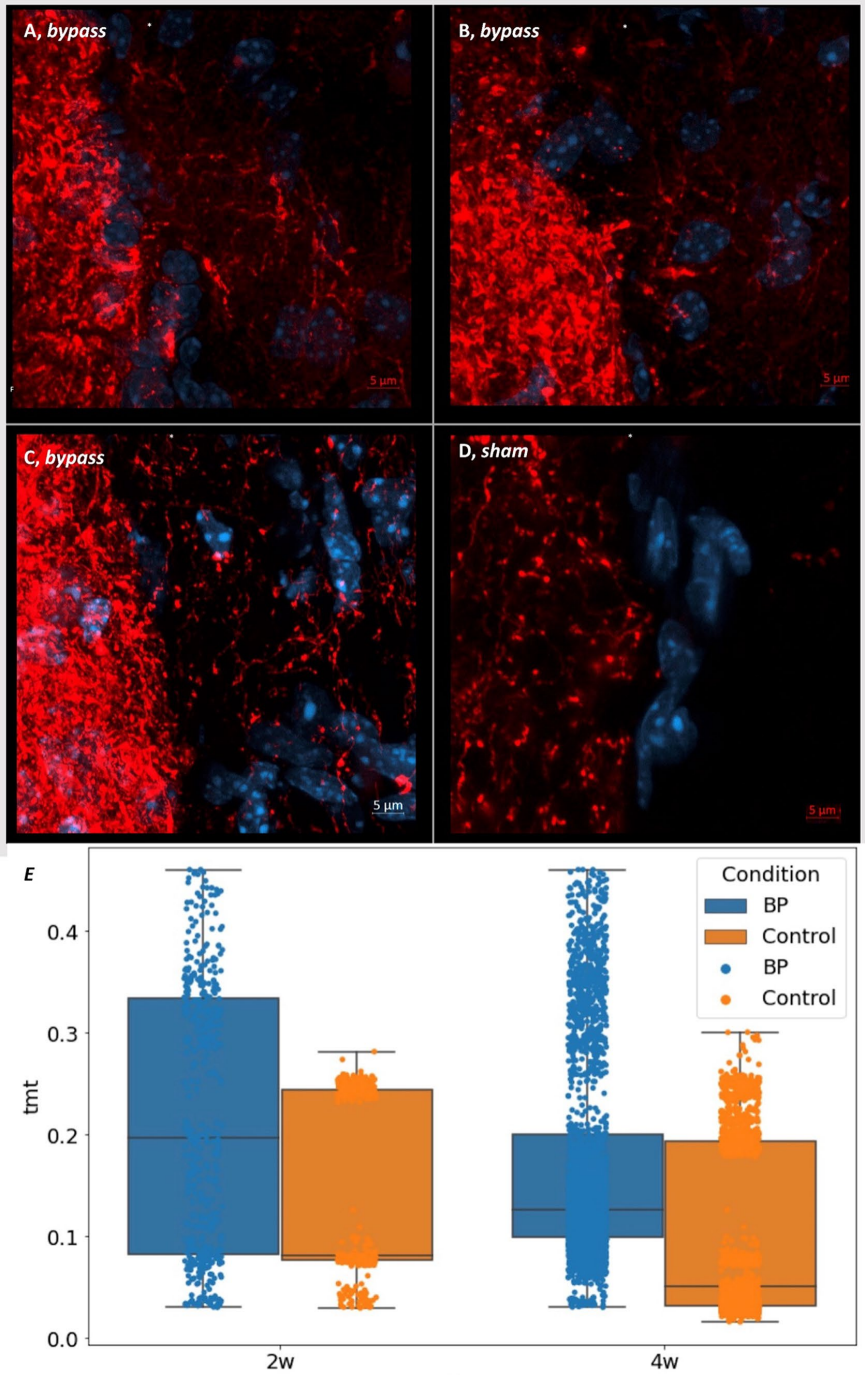
High magnification (100X) images if the interhemispheric fissure shows individual neurite crossings after the BP surgery. (**A**), (**B**) and (**C**) represent three different BP mice sacrificed four weeks after surgery, representing the changes seen after surgery at this time. Red signal depicts tdTomato from AAV injections in one hemisphere as shown in Fig. 3. Blue shows DAPI signal. (**D**) As in A, but shows a sham example, illustrating no interhemispheric crossings visible. (**E**) The tdTomato (tmt) signal normalized to the injected cortex was significantly higher in bypass animals at both time points (n = 5 for each group). Each dot represents a portion of the analyzed region around the IH fissure. Statistical differences were calculated using each animal as a single data point. The physical midline is marked with an asterisk.

Single neurite crossings were observed in three different BP mice, with high-magnification images revealing neurites containing multiple synaptic boutons spanning the newly formed interhemispheric bridge (Fig. 4A-C). In contrast, sham-operated animals lacked these neurite crossings and synaptic formations (Fig. 4D).

The tdTomato fluorescence intensity was measured on the left (non-injected) hemisphere and normalized using the fluorescence on the right (injected hemisphere). BP mice had a higher corrected intensity than sham at 2 (0.28 ± 0.16 BP vs 0.13 ± 0.12 sham, p < 0.05, Mann–Whitney U test, n = 5 per group) and 4 weeks (0.16 ± 0.01 BP vs 0.14 ± 0.1 sham, p < 0.05, Mann–Whitney U test, n = 5 per group) after surgery (Fig. 4E).

## Discussion

The adult brain is capable of re-purposing cortical and subcortical areas in response to injury. For example, subcortical reorganization allowed Asboth et al*.* to create a brain stem bypass to treat spinal cord injury in rodents, using the rubro- and vestibulospinal tracts for voluntary motion^25^. Furthermore, cortical regions are capable of cross-modal reorganization by subcortical reorganization, synaptic unmasking, axonal sprouting, or a combination of these^26–30^. The potential outcome of surgically connecting adjacent gyri through a bridge is not yet known, but this could link neurons located in different gyri that are in close spatial proximity, potentially allowing for the rerouting of information and bypassing damaged white matter tracts (Fig. 1A).

To explore the effect of a cortico–cortico transpial bypass in a gyrencephalic model, we sought to assess the effect of removing the pia between brain hemispheres in mice. Pial removal did not lead to functional impairments or decreased thickness of cortical layer I. Instead, we found remodeling of the subpial glial structures and allowed for interhemispheric neurite growth through the newly formed bridge. When the pia was removed, the brain parenchyma herniated through the pial window and physical contact between cortices was established without the need to use a scaffold or suturing the adjacent pia. Our model presents some limitations. Although we showed neurite sprouting occurs after pial removal, since mice are lissencephalic, it does not account for its potential functional results in a folded brain. Joining homotopic cortical regions may result in a favorable environment to form new connections, which may not be present when different types of neurons from adjacent gyri interact. Finally, thinning of cortical layer I might be the result of direct surgical injury, or secondary to devascularization from the removal of pial blood vessels.

Axons are highly dynamic; they can grow and form new connections in the adult brain in response to injury^31^. They grow and find targets by incorporating attractive and repulsive signals within their microenvironment. Axonal growth spontaneously occurs in the adult brain after stroke, and sprouting in this setting leads to functional recovery^22,32^. Our model induces interhemispheric neurite growth, likely by inducing a controlled injury of the superficial cortex and pia. After bypass we observed a parenchymal bridge between the hemispheres and no pial barrier. Therefore, we show there is no ‘stop’ signal at the IH fissure, and thus, neurites can span across hemispheres.

Here, we present data that shows mice can form new connections between brain hemispheres after pial bypass. Future directions include functional tests of these interhemispheric sprouting neural fibers using calcium imaging, to explore longer time points after surgery, and combine the bypass operation with subcortical injury to functionally interrogate the model. Furthermore, it would be interesting to explore if cortico–cortico transpial bypassing can be enhanced. Adopting different stimulation protocols such as electric, optogenetic, pharmacological, or behavioral (i.e., physical tasks or cage enrichments), could induce the use of this bridge. These steps can be accomplished in mice after which, a gyrencephalic model would be needed to assess the effect of intergyral cortical transpial cortical connection.

## Electronic supplementary material

Below is the link to the electronic supplementary material.

Supplementary Material 1

## Data Availability

The datasets generated during and/or analyzed during the current study are available from the corresponding author upon reasonable request.
