# Supplementary material for: Interhemispheric axonal sprouting occurs after pial removal in mice
Source: Sci Rep. 2024 Oct 21;14:24765. doi: 10.1038/s41598-024-75278-4 (PMC11494079; doi:10.1038/s41598-024-75278-4)
Supplement: Supplementary file 1 — Supplementary Material 1 [file 41598_2024_75278_MOESM1_ESM.pdf]

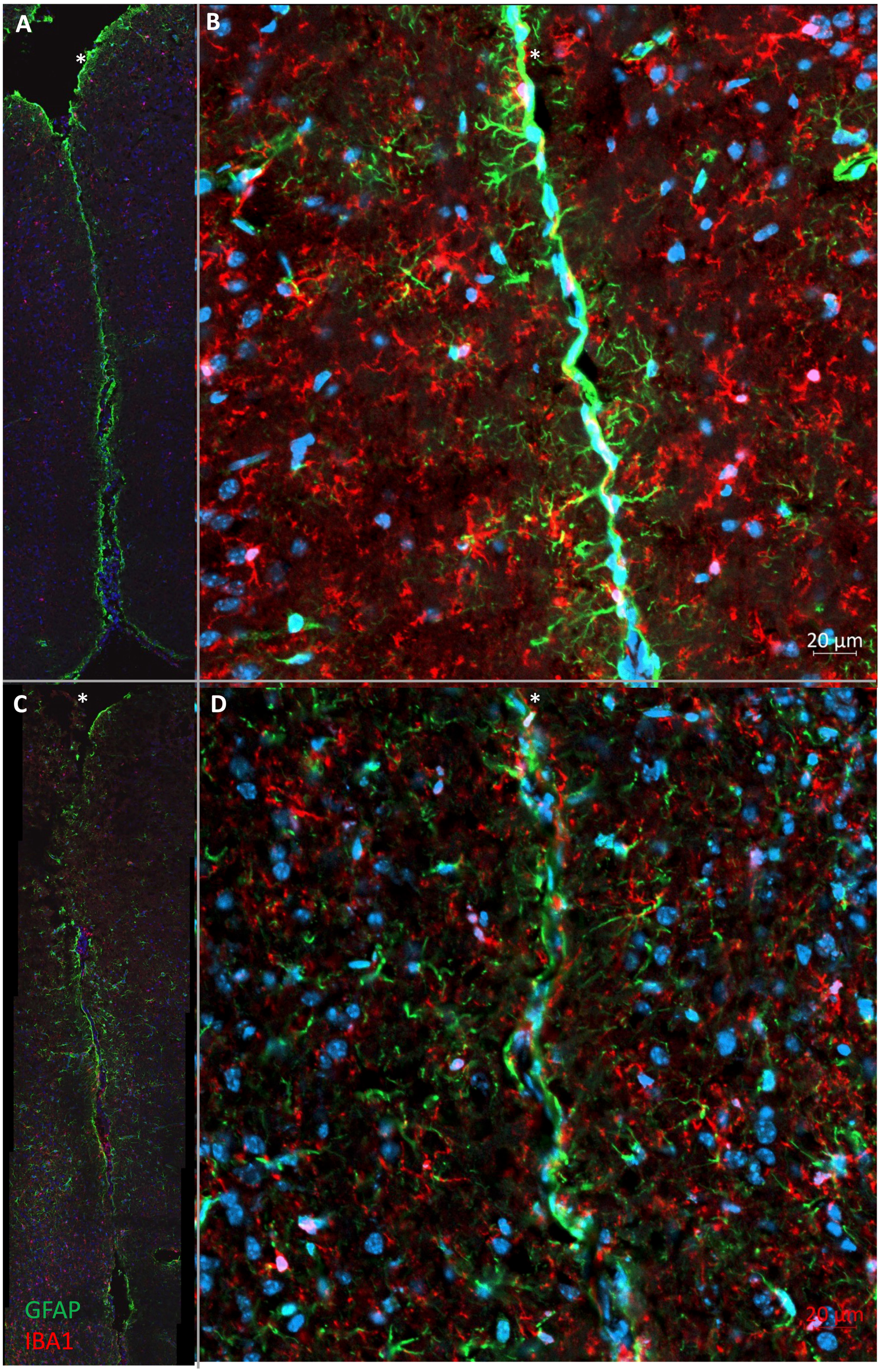

Supplementary Figure 1. Low and high magnification showing physical continuity between Sham and BP cortices. A and **B** depict low and high-magnification images of the interhemispheric fissure in Sham mice, whereas **C** and **D** show analogous images for a bypass mouse. In both conditions, there is physical continuity between the right and left hemispheres. A straight interhemispheric fissure in **A** and **B** is replaced with a sinuous one in **C** and **D**, while maintaining intercortical contact, forming a tissue bridge. Gfap in green, Iba1 in red and DAPI in blue.

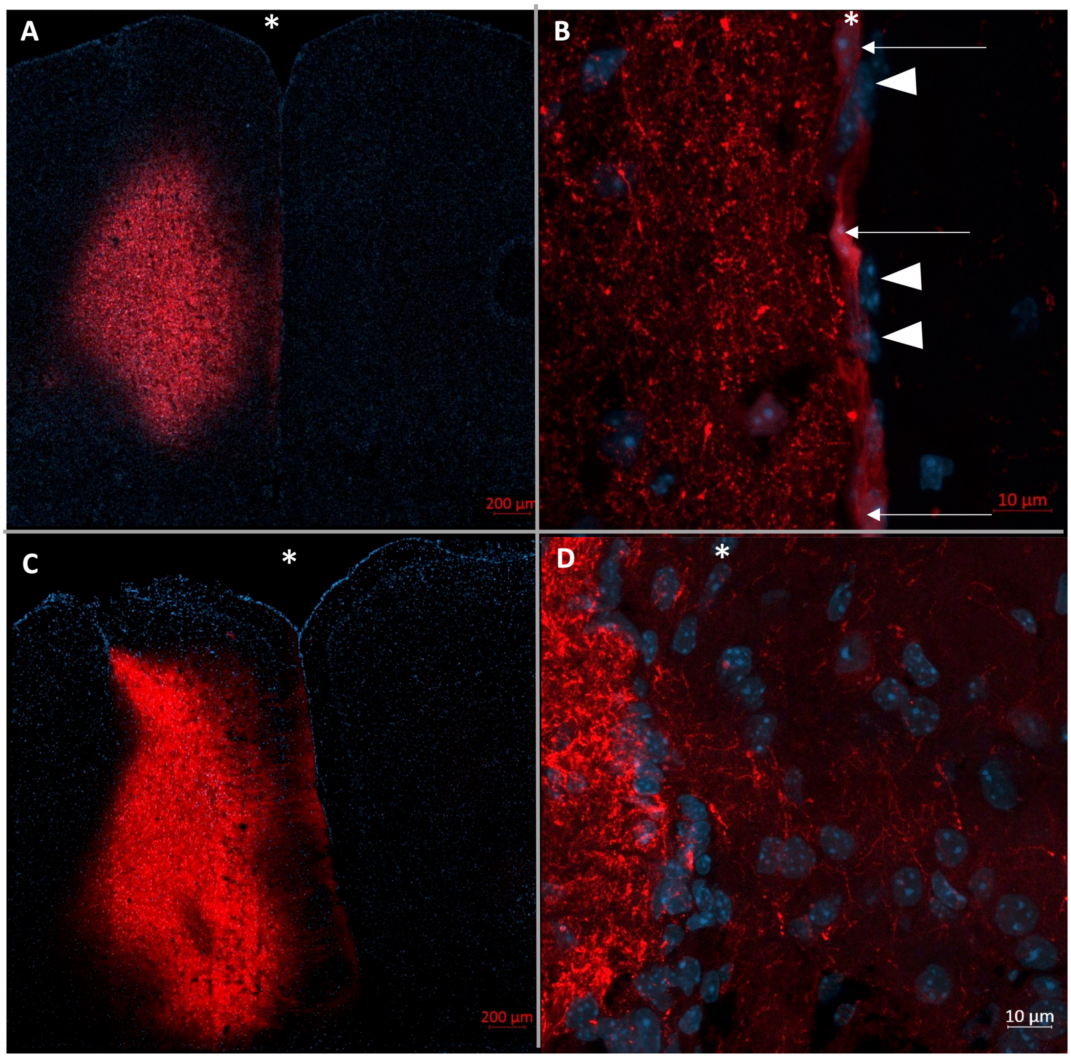

Supplementary Figure 2. AAV carrying a universal promoter transduces neurons and pial cells marking the interhemipsheric pia in Sham but not in bypass mice. A and **B** show a low and high magnification depicting tdTomato-positive signal in the pial lining of the ipsilateral cortex (arrows), the contralateral pial nuclei are tdTomato-negative (arrowheads). **C** and **D** show analogous images in a bypass mouse; in this case, both the tdTomato-positive pial cells and contralateral nuclei are lost, signaling the effective removal of both pial layers.
